# Comparative effectiveness of daptomycin versus vancomycin among patients with methicillin-resistant *Staphylococcus aureus* (MRSA) bloodstream infections: A systematic literature review and meta-analysis

**DOI:** 10.1371/journal.pone.0293423

**Published:** 2024-02-21

**Authors:** Yau Adamu, Mireia Puig-Asensio, Bashir Dabo, Marin L. Schweizer

**Affiliations:** 1 Department of Pharmacology and Therapeutics, Faculty of Pharmaceutical Sciences, Bayero University, Kano, Nigeria; 2 Department of Internal Medicine, Carver College of Medicine, University of Iowa, Iowa City, Iowa, United States of America; 3 College of Public Health, University of South Florida, Tampa, Florida, United States of America; 4 Department of Medicine, School of Medicine and Public Health, University of Wisconsin, Madison, WI, United States of America; University of Tripoli, LIBYA

## Abstract

**Background:**

In the treatment of methicillin-resistant Staphylococcus aureus (MRSA) bloodstream infections (BSIs), vancomycin stands as the prevalent therapeutic agent. Daptomycin remains an alternative antibiotic to treat MRSA BSIs in cases where vancomycin proves ineffective. However, studies have conflicted on whether daptomycin is more effective than vancomycin among patients with MRSA BSI.

**Objective:**

To compare the effectiveness of daptomycin and vancomycin for the prevention of mortality among adult patients with MRSA BSI.

**Methods:**

Systematic searches of databases were performed, including Embase, PubMed, Web of Science, and Cochrane Library. The Newcastle Ottawa Scale (NOS) and Revised Cochrane risk-of-bias tool for randomized trials (RoB 2) were used to assess the quality of individual observational and randomized control studies, respectively. Pooled odd ratios were calculated using random effects models.

**Results:**

Twenty studies were included based on *a priori* set inclusion and exclusion criteria. Daptomycin treatment was associated with non-significant lower mortality odds, compared to vancomycin treatment (OR = 0.81; 95% CI, 0.62, 1.06). Sub-analyses based on the time patients were switched from another anti-MRSA treatment to daptomycin demonstrated that switching to daptomycin within 3 or 5 days was significantly associated with 55% and 45% decreased odds of all-cause mortality, respectively. However, switching to daptomycin any time after five days of treatment was not significantly associated with lower odds of mortality. Stratified analysis based on vancomycin minimum inhibitory concentration (MIC) revealed that daptomycin treatment among patients infected with MRSA strains with MIC≥1 mg/L was significantly associated with 40% lower odds of mortality compared to vancomycin treatment.

**Conclusion:**

Compared with vancomycin, an early switch from vancomycin to daptomycin was significantly associated with lower odds of mortality. In contrast, switching to daptomycin at any time only showed a trend towards reduced mortality, with a non-significant association. Therefore, the efficacy of early daptomycin use over vancomycin against mortality among MRSA BSIs patients may add evidence to the existing literature in support of switching to daptomycin early over remaining on vancomycin. More randomized and prospective studies are needed to assess this association.

## Introduction

Methicillin-resistant *Staphylococcus aureus* (MRSA) infections continued to be a significant public health challenge in the United States, with reported mortality ranging from 20% to 30% [1, 2]. Vancomycin has been the first-line antibiotic for the treatment of MRSA infections, particularly bloodstream infections (BSIs) [3]. However, the available evidence demonstrates challenges regarding its safety profile as well as tissue penetration and slow killing time [4, 5]. Clinical failures in vancomycin treated MRSA patients have been associated with strains of MRSA that are less susceptible to vancomycin as measured by higher vancomycin minimum inhibitory concentrations (MICs) [6]. Alternative antibiotics to treat MRSA BSI are recommended when vancomycin MIC is greater than 2mg/L [6–8].

Daptomycin, a lipopeptide antibiotic, is one of the alternative antibiotics recommended for treatment of MRSA BSI with high vancomycin MIC and treatment failure [8]. However, the use of daptomycin as an alternate anti-MRSA antibiotic is limited by issues associated with cost and antibiotic stewardship [9–11]. The use of daptomycin to treat MRSA BSIs in situations of vancomycin treatment failure has been increasing [12]. Moreover, the recent approval of generic daptomycin by the Food and Drug Administration (FDA) may lower the cost of daptomycin, leading to increased frequency of daptomycin use [13]. The cost of daptomycin treatment may also be comparable to vancomycin considering that vancomycin therapy requires AUC and trough-based therapeutic drug monitoring [14, 15]. A survey conducted among infectious disease physicians showed that 71% of the participating physicians used daptomycin to treat at least one MRSA BSI patient each year [16].

Several studies have been published comparing the effects of daptomycin versus vancomycin on preventing all-cause mortality in MRSA BSI patients. The inconsistency of the available results does not provide clear guidance to physicians on when to use daptomycin and when to switch from vancomycin to daptomycin for optimal treatment of MRSA BSI. The objective of this study was to compare the effectiveness of daptomycin versus vancomycin for the prevention of mortality, clinical failure and persistent bacteremia among adult patients with MRSA BSIs.

## Materials and methods

### Protocol development

This meta-analysis was conducted and reported according to the Preferred Reporting Items for Systematic Reviews and Meta-Analyses (PRISMA) and Meta-analysis Of Observational Studies in Epidemiology (MOOSE) guidelines [17, 18]. In addition, the study’s research question was formulated following the Population, Intervention, Comparator, and Outcomes (PICO) model, where the population of interest were adult patients with MRSA BSI, the Intervention/Exposure was defined as daptomycin use either initially or switch from vancomycin, and the comparator was vancomycin [19, 20]. The primary outcome was all-cause mortality. Mortality was defined as all-cause mortality measured after MRSA infected patients were followed-up for differing lengths of time, including in-hospital, 14-days, 30-days, 42-days, or 60-days after at least 48 hours of vancomycin or daptomycin therapy. The secondary outcomes were clinical failure and persistent bacteremia. Clinical failure as defined by the included studies. These definitions included a composite of all-cause mortality, 7-day clinical or microbiologic failure, failure at end of treatment (EOT), MRSA BSI relapse, new or worsening signs and symptoms of infection while receiving MRSA therapy, failure to eradicate the organism from the bloodstream at the end of at least 7 days of primary therapy, and treatment switch due to poor evolution or death during treatment. Persistent bacteremia as defined by included studies and included persistent positive MRSA blood cultures ≥5 days after the start of drug of interest (vancomycin or daptomycin) or from index blood culture during therapy, positive MRSA blood cultures within 14 days before cessation of therapy, and positive MRSA blood cultures ≥7 days after diagnosis while receiving effective treatment for ≥ 5 days.

### Search strategy

**Fig 1:**
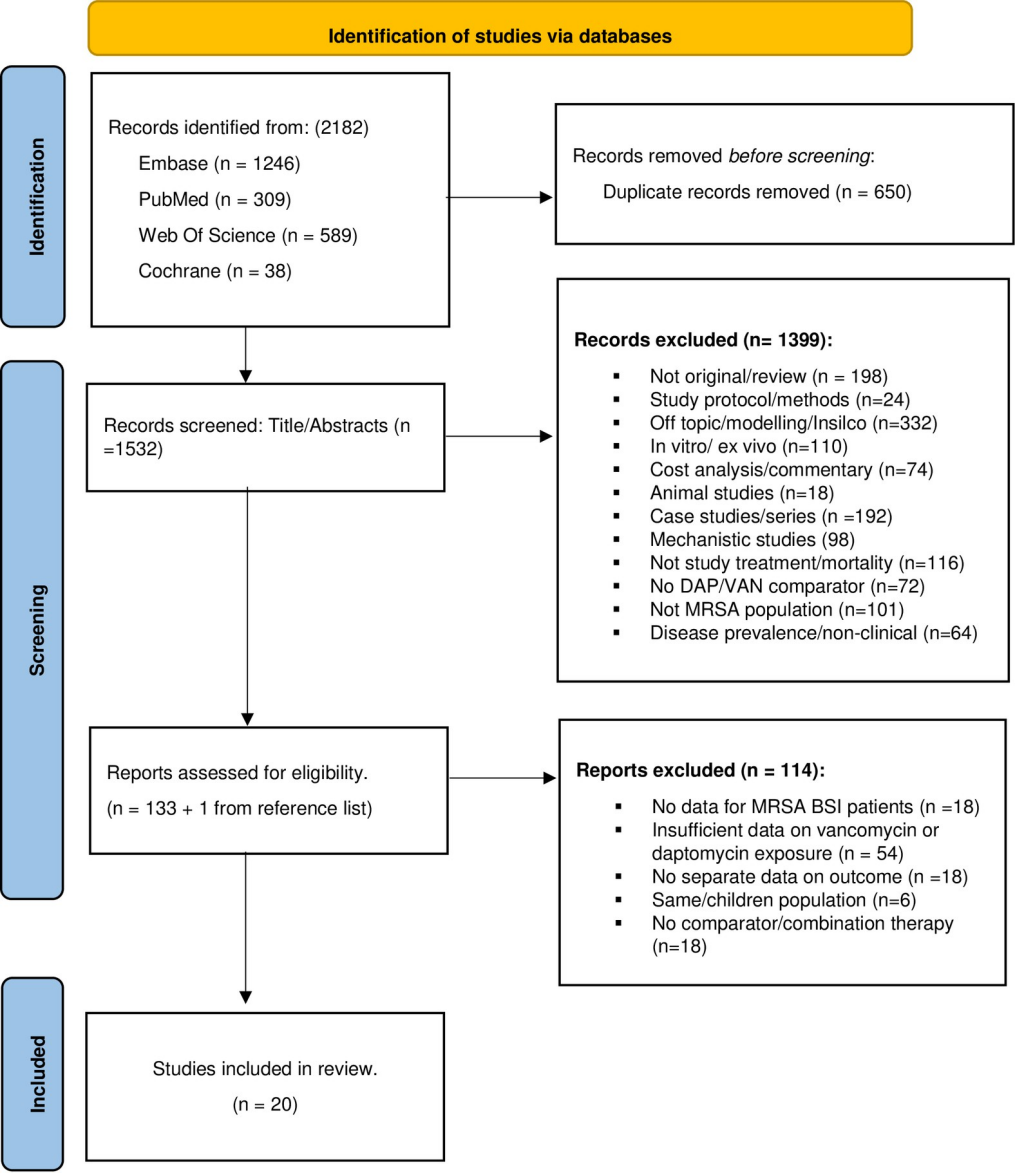
A PRISMA diagram showing the study selection process.

A systematic electronic literature search was conducted into the PubMed, EMBASE, Cochrane Library, and Web of Science databases from their inception up to the **7**^**th**^ **of July 2023**. The bibliographies of the included studies were examined to identify additional studies. A search strategy was conducted using the following terms without language restriction to find articles that were relevant to this study; ‘Vancomycin’ AND ’methicillin resistant *Staphylococcus aureus’* AND ‘Daptomycin’. Study selection was conducted based on *a priori* inclusion and exclusion criteria. Authors were contacted to retrieve additional information not published in the original article. The inclusion criteria were Randomized Control Trials (RCTs), cohort, and case control studies reporting information on mortality comparing daptomycin versus vancomycin use in adult patients with MRSA BSI. The detailed exclusion criteria are listed in Fig 1. Briefly, excluded studies were case reports, case series, studies without sufficient information on the exposure of interest, comparator, or primary outcome among patients with MRSA BSI.

### Study selection and data collection

The titles, abstracts and full-text articles were screened for inclusion. The abstracted data from selected articles included: 1) first author and year of publication; 2) study design and study duration; 3) population characteristics; 4) country in which the study was conducted; 5) exposure characteristics; 6) presence of MRSA-associated endocarditis; 7) end point of assessment including mortality, persistent bacteremia, and clinical failure; and 8) confounders adjusted for in the study (Fig 1). These data were collected independently by two authors (YA and BD) using data abstraction forms. The disagreements were resolved in meetings by consensus. When multiple studies described the same population, the study with the most details and the lowest risk of bias was included. Where one study reported different mortality rates based on follow-up duration (i.e., in-hospital vs 30-days vs 60-days), the shortest duration of follow-up was included [21].

### Quality assessment

The quality of individual observational studies was assessed using the Newcastle Ottawa Scale (NOS) based on selection, comparability, outcomes in cohort studies, or exposure in case-control studies. Studies with quality scores of 6 or more were classified as moderate to high quality publications [22]. RCTs were assessed using the Revised Cochrane risk-of-bias tool for randomized trials [23].

### Data analysis

The pooled odd ratios with corresponding 95% CIs were calculated using random effects models. To explore the source(s) of heterogeneity and robustness of the study findings, subgroup analyses were performed based on the study design, duration of follow-up, vancomycin MIC and switch time from vancomycin to daptomycin. Also, sub-group analyses were conducted based on patients with endocarditis, and patients treated with other additional anti-MRSA antibiotics. Statistical heterogeneity was measured using the *I*-square statistic test. Publication bias was investigated using R functions, “regtest” and “ranktest” to perform Egger’s regression test for funnel plot asymmetry using RStudio. The data presented were analyzed using Excel, RevMan software version 5.4.1 and Rstudio.

## Results

### Study characteristics

Twenty studies out of the 2,182 articles identified were included in the meta-analysis (Fig 1). Nine were matched retrospective cohort studies, six were unmatched retrospective cohort studies, one case-control study, one combined prospective and retrospective study designs, and one quasi-experimental study. The remaining two were RCTs. The characteristics of the studies included are summarized in Table 1.

**Table 1:** Main characteristics of the included studies.

Author, Year	Study design, setting (study period), and location	MRSA Population Characteristics				Exposure Characteristics			Endpoint of Assessment			Confounders Adjusted	
		Sample size (DAP vs VAN groups)	Sex and race. Special traits of study population (if applicable)	VAN MIC (mg /L) (Test Method)	Patients with MRSA ED (DAP vs VAN)	VAN (Trough levels)	DAP (Dose)	Use of concomitant anti-MRSA antibiotics (DAP vs VAN)	Mortality	Persistent bacteremia	Clinical failure		Reason for switching to DAP. Time of switching
Moore 2012	Retrospective case-control at a single center (2005–2009) USA	177 (59 vs 118)	Mixed sex and race patients ≥ 73% African American Pneumonia source excluded	Range MIC: >1 and ≤2mg/L (E-test)	17 (29%) vs 34 (29%)	Started on VAN within 48 hours of diagnosis and received for ≥ 48 hours. Median trough levels 18 mg /L	Started within 48 hours or switched from other treatment within 14 days after diagnosis: Median dos: 7mg/kg/day	Received an aminoglycoside or rifampin for ≥3 days: 22 (37%) vs 60 (59%)	60-day mortality	Positive MRSA blood cultures ≥ 7 days from index blood culture, during therapy (or after starting DAP)	Composite Clinical Failure^α^	Matched on age, APACHE-II, score, and risk level of infection source	Worsening situation: 60%, adverse event: 3%, unknown: 38%. Median time to switch: **5 days (IQR 3–9 days)**
Moise 2016	Retrospective matched cohort at Multiple center (2005–2012) USA	170 (85 vs 85)	Mixed sex patients. No Data on race. Tunneled- catheter-related bacteremia or pneumonia sources excluded	Range MIC: 1.5 to 2 (60% of isolates had VAN MICs of 2). (E-test, Microscan or Phoenix)	20 (24%) vs 11 (13%)	Received an anti-MRSA antibiotic within 72 hours of diagnosis and VAN continued for ≥ 72 hours. Median trough levels 17 mg /L	Received anti-MRSA within 3-days of diagnosis & continued for ≥ 72 hours. Median dose 6 mg/kg/day (≥ 8 in 26% of patients)	Received an aminoglycoside or rifampin: 24 (28%) vs 23 (27%)	60-day mortality	Positive MRSA blood cultures ≥ 5 days	Composite variable^β^	Patients matched on age, ICU status, and infection source	83% because of a VAN MIC of 2 and 17% because of a VAN MIC of 1.5 **Early daptomycin (≤5 days)**
Schweizer 2021	Retrospective cohort, Multiple acute care VA hospitals, (2007–2014), USA	7411 (606 vs 6805)	Mainly males (> 97%) and mix race patients	No MIC restriction. MIC ≥2: 16% in DAP and 8% in VAN groups	Endovascular infection; 30%, vs 18%	Initial start with VAN Median trough levels: NR	Switched to DAP early 3 day or any time. DAP dose: 93% given≥5mg/kg/day & 7% lower.	Other anti-MRSA antibiotics) 4.5% vs 1.2%	30-day mortality after first receipt of VAN	No data	No data	^b^Demographic^s^, patient characteristics, facility complexity, IDPC	Reason for switching NR*. Time to switch within 3-days and switch anytime.
Kalimuddin 2018	RCT at a single tertiary-care hospital, (2014–2015), Singapore	14 (7 vs 7)	Mixed sex, Asian patients	Range MIC: ≥ 1.5 to < 2 (E-test or VITEK™-2 system)	No patients with endocarditis	Started on VAN at ≥ 48 hours after MRSA BSI diagnosis. VAN adjusted to achieve trough level = 15–20 μg/L	Started on DAP and continued for at least 72 hours. DAP dose of 6 mg/kg/day (8 if complicated)	No other anti-MRSA antibiotics	60-day all-cause mortality	Positive MRSA blood cultures ≥ 7 days from index blood culture	No data	Randomization	NA (not applicable)
Arshad 2017	Retrospective matched cohort, A tertiary-care hospital (2009–2013), USA	102 (46 vs 56)	Mixed sex and race patients	MIC ≥1.0 (Broth dilution E-test)	19 (41%) vs 13 (23%)	Not clear—selection of antibiotics was at the IDP discretion. No data on dose	Not clear—the selection of antibiotics was at IDP discretion. No data on dose	NR	30-day mortality	NR	Composite failure^δ^	Matched based on age, ICU status, disease severity.	Reason for switching NR. No statement about initial use or witch
Murray, 2013	Retrospective matched cohort at a single tertiary-care hospital, (2005–2012) USA	170 (85 vs 85)	Mixed sex patients. Data on race not reported. Catheter-related bacteremia or pneumonia sources excluded	Range: > 1 and ≤2 (94% of isolates had VAN MICs of 2) (Microscan or E-test)	20 (24%) vs 20 (24%)	Started on VAN within 48 hours after MRSA BSI and continued for > 72 hours. VAN adjusted to achieve trough of 15–20 mg/l. (median 17.6)	Most patients switched to DAP within 3 days. On DAP for > 72 hours. DAP median dose administered 8.4 mg/kg/day	Received an aminoglycoside or rifampin: 26 (31%) vs 40 (47%)	30-day mortality	Positive MRSA blood cultures ≥ 7 days from index blood culture	Composite clinical failure including 30-day mortality or persistent bacteremia	Matched based on age, Pitt bacteremia score, and primary source of bacteremia	DAP: 92.9% patients were switched once VAN MIC > 1 was identified (only 6 patients started on DAP): Early switched to daptomycin within 3- days
Claeys, 2016	Retrospective matched cohort at three hospitals, cohort, (2010–2015), USA	262 (131 vs 131)	Data on sex and race not reported. Pneumonia source excluded	MIC of VAN >1 mg /L (59.5% of ATS test & 5.7% of BMD tested had MIC of 2)	25 (15%) vs 25 (15%)	VAN for at least 72 h of MRSA-directed therapy Median trough levels through levels 17.7 mg /L	Most patients switched to DAP within 3-days (81% of cohort) & remain for ≥72 hrs. DAP median dose 8.2 mg/kg/day)	24% vs 21% (mostly used ceftaroline or rifampin)	30-day all-cause mortality	Positive MRSA blood cultures ≥ 7 days	Composite clinical failure^ε^	ICU admission, infection sources, AKI, IE, Source control. Propensity matching, AOR	Reason for switching NR; Switch within 72 hours
Weston, 2014	Retrospective matched cohort at a single tertiary-care hospital, (2001–2011), USA.	150 (50 vs 100)	Mixed sex and race patients. >70% white patients Pneumonia source excluded	No MIC restriction MIC ≥2: 20% in DAP and 22% in VAN group (BM)	13 (26%) vs 11 (11%)	Patients had VAN for at least 3 consecutive days. VAN adjusted to achieve trough level of 10–20 mg/L or 15–20 mg /L from 2009	Switched from an active anti-MRSA antibiotic (82% VAN) to DAP within 10 days. Recommended DAP dose: ≥6 mg/kg/day	NR	In hospital mortality	Positive MRSA blood cultures ≥5 days after the start of drug of interest (VAN or DAP)	Composite Clinical failure^η^	Matched, GFR, IE, liver disease, source of infection, and duration of treatment/ MRSA therapy	Reasons for switch: -26% persistent blood cultures, - 22% decided by IDP, -14%clinical failure, & -12% unknown Switched within 10 days
Cheng, 2012 Abstract	Retrospective cohort at a single hospital, (2009–2010), Taiwan	78 (26 vs 52)	Data on sex and race not reported.	MIC ≥ 1.5 (E-test)	NR	Started on VAN and continued for ≥72 hours. Loading dose 25–30 mg/kg then 15–20 mg/kg 12 hourly.	Switched to DAP within 5 days of diagnosis and continued for ≥72 hours. DAP dose 8–10 mg/kg/day.	NR	30-day mortality	Positive MRSA blood cultures ≥7 days after diagnosis and despite effective treatment for ≥ 5 days	Clinical failure (persistence of clinical features or MRSA bacteremia)	Matched patients based on sex, age, and Pittsburgh bacteremia score –	Reason for switching NR; Early switch within 5 days.
Fowler, 2006 and Rehm et al., 2008	Subset analysis of an open label RCT at Multicenter, (2002–2005), USA	88 (45 vs 44)	Mixed sex and race patients	No MIC restriction. No specific data reported.	13 (29%) vs 10 (23%). Right sided IE	Started on VAN within < 48 hours of diagnosis and continued for 72 hours. VAN dose 1 g 12 hourly	Started with DAP within < 48 hours of diagnosis and continued for ≥ 72 hours. DAP dose 6 mg/kg/day	Received gentamicin for the first 4 days: 0% vs 91%	42-day mortality	Microbiologic failure^γ^	No response to the study drug based on ongoing signs and symptoms of infection	Randomized, gentamycin given to 107 of 115 standard therapy arm vs 1 patient in DAP group	NA
Gaudard, 2019	Retrospective cohort at a single center, (2010–2012) France	7 (4 vs 3)	No data on sex and race. Included only cardiovascular ICU patients with PSI & B	NR	NR	Had VAN within ≥ 48 hours culture. Dose: 30 mg/kg in 1 h. adjusted to 15–30 mg /L. TDM to maintain 20–30 mg /L through.	Received at least 2 doses of DA. Dose: 10 mg/kg/day	Not given other anti-MRSA	28-day and 180-day mortality	No data	No data	No adjustment in the assessment of mortality	Some switched but not reported reason and time for switching. Time to switch: NR
Carugati, 2013	Prospective cohort from multiple countries, (2008–2010), (E, NA&A)	25 (7 vs 18)	Mixed sex patients. Data on race not reported.	NR	100% of left-sided endocarditis in both study groups	Received VAN during >50% of antibiotic duration as recommended by the AHA. No dose data	Received DAP during >50% of recommended antibiotic duration. Median dose 9.2 mg/kg/day	Combo therapy 57%* vs NR. Combo used: DAP +rifampin (2),—DAP + Fosfomycin (1)—DAP + levofloxacin (1)	In-hospital and 6-month mortality	Positive MRSA blood cultures > 72 hours after targeted antibacterial treatment	NR	NR	Reasons for switched: clinical failure, PB, adverse events, unknown. S**witched** to DAP in 19 (67.9%): Time to switch NR
Barlow, 2021	Retrospective cohort at single tertiary-care hospital, (2015–2019), USA	53 (10 vs 43)	Mixed sex patients Data on race not reported. Pneumonia source excluded	MIC < 1 mg/dL in all VAN cohort versus 92.3% in the DAP cohort (NR)	12 (27.9%) vs 4 (30%) of DAP	Received VAN at least 72 hours. 11 patients (26%) switched to DAP as outpatients. Median through levels of 15.4	Received DAP at least 72 hours. Median DAP dose 6 mg/kg/day	Aminoglycoside or rifampin in 2 (15%) of DAP vs 7 (16%) of VAN cohorts. No patient given a combo of Anti-MRSA for ≥ 72 h	In-hospital and 30-day mortality	Positive MRSA blood cultures for > 5 days	NR	Adjusted for clinical failure: adverse events, infectious complications experienced	Reason for switching NR (26% switched as continuation outpatient therapy) The majority median time to switch: 2 (IQR 1, 2.25) days
Maeda, 2016	Retrospective cohort at a tertiary-care hospital, Japan (2009–2014)	92 (5 vs 87)	Mixed sex patients Data on race not reported	MIC 1–1.5 (MDS)	8 (8%) of entire cohort	Started on VAN as the first given antibiotic agent. No dose data	DAP started as the first-line antibiotic agent MRSA. No dose data	NR	30-day mortality	NR	NR	No adjustment in the assessment of mortality	NA
Ruiz et al., 2018	Retrospective cohort at a tertiary-care, (2010–2015), Spain	21 (7 vs 14)	Mixed sex patients Data on race not reported	VAN. MIC >1 in 67.1% of entire cohort (AMV)	NR	First-line treatment No dose data	First-line treatment No dose data	NR	30-day mortality	NR	Treatment failure^β^	Multivariate logistic regression: ICU admission, sources of infections	NA
López-Cortés, 2012 Abstract	Prospective & retrospective cohort, Multicenter (2008–2011), Spain	112 (39 vs 73)	Mixed sex patients. Data on race not reported	MIC > 1 (E-test)	NR	VAN during the first 5 days, and for >50% of the therapy duration. No dose data	DAP in first 5 days, & for >50% of the duration of therapy. No dose data	NR	14-day and 28-day mortality	Positive MRSA blood cultures for > 3 days	Therapeutic failure^π^:	Age, Pitt score, source, severity of SIRS, MIC, therapy, complications.	Reason for switch therapy: probably poor evolution infection. Time to switch: NR
Usery, 2015	Retrospective cohort at a tertiary-care hospital, USA (2008–2010).	107 (53 vs 54)	Mixed sex patients. > 72% African American	VAN MIC ≥2 in 60% in DAP vs 33% in VAN) (MicroScan)	6 (11%) vs 6 (11%)	Had VAN ≥ 7 days. An average VAN dose of 13.6± 4 mg/kg/day	Received DAP ≥ 7 days. Initial dose of 6.7 ±1.8 mg/kg/day	Exclusion of patients treated with > 1 anti-MRSA agent	All-cause mortality	Positive MRSA blood cultures within 14 days before therapy ending.	Clinical failure/ cure^ρθ^	74% had IDPC: (29/54 VAN, 51/53 DAP, and 4/15 linezolid)	92% of patients received VAN before DAP, but reason for switching: NR Time to switch NR
Kullar, 2013	QE at a single trauma center (2005–2007) vs. (2008–2010), USA	170 (100 vs 70)	Data on sex and race not reported	MIC > 1 (E-test)	20% vs 30%	Empiric VAN ≥ 1 mg/L. Targeted trough levels of 10–20 (2005–06) and 15–20 mg /L during 2008–2010	Switched to DAP after a median of 3 days. Recommended DAP dose ≥6 mg/kg/day	20.0% (mainly gentamicin and rifampin) vs 31.4% (mainly gentamicin)	In-hospital mortality	Positive MRSA blood cultures for ≥ 7 days	Treatment failure ^λ^	Subset analysis	Switched if the isolate had a VAN MIC >1 Median time before switched to DAP: 3 days (IQR 2–3 days)
Nichols, 2021	Retrospective cohort at a tertiary hospital, USA (2011–2019)	32 (15 vs 17)	Mixed sex and race patients. >82.14% white	MIC 0.5–2 mg/L (MicroScan and Vitek (Biomerieux)	NR	Switched from combination therapy/first line treatment. No dose data	Switched from combination therapy/first line treatment. No dose data	All monotherapy as either switched from combination therapy/First-line treatment	Inpatient infection-related mortality	Bacteremia recurrence within 60 days	A composite clinical failure	NR in the assessment of outcomes in DAP vs VAN monotherapy groups.	Switched from combination therapy/First-line treatment. Time to switch: NR
Carroll 2022	Retrospective cohort at a tertiary hospital, USA (2014–2021)	55 (48 vs 7)	Not stated	Not stated	Not stated	Stated DAP/CFT combination and de-escalated to VAN alone. No dose data	Stated DAP/CFT combination and de-escalated to DAP alone. No dose data	All monotherapy as switched from combination therapy	Inpatient infection-related mortality	Persistent Bacteremia recurrence	NR	Adjusted for co-morbidities and source control	Switched from combination therapy/First-line treatment. Time to switch: NR

### Study population

Twenty studies reported the effectiveness of daptomycin compared with vancomycin in terms of mortality. Of the 9,523 adults with MRSA BSI, 1,527 (16.03%) and 7,996 (83.97%) were treated with daptomycin and vancomycin, respectively. The age range of the patients was 21 to 91 years. The majority of studies included MRSA strains with vancomycin MIC >1 mg/L, four studies enrolled only patients infected with MRSA BSI strains with vancomycin MIC between 1 and 2 mg/L [24–27]. However, 50% to 94% of strains in three studies had vancomycin MIC above 2 mg/L and 16% to 20% of strains from two studies had vancomycin MIC ≥2 mg/L [27–31]. Among the sixteen studies with available data, the percentage of endocarditis patients from individual studies ranged from 2.9% to 100% (Table 1). One study excluded all patients with endocarditis [24], while another study exclusively recruited endocarditis patients [32].

### Exposure and outcome assessment

Receipt of daptomycin was defined as either the initial receipt of daptomycin treatment or switching from another anti-MRSA treatment to daptomycin and continued daptomycin for at least 72 hours. Four studies evaluated the initial receipt of daptomycin without switching [24, 25, 33, 34]. The remaining observational studies evaluated patients who were switched to daptomycin within 3 days, within 5 days, or within 10 days [26–30, 35–38]. Some studies mentioned switching to daptomycin at any time or after five days [27, 28, 36–38]. One study had no statement about initial use or switching time [39]. Reasons for switching to daptomycin are reported in **Table 1**. Most studies administered the recommended daptomycin dose of 6 mg/kg/day and increased it to 8–10 mg/kg/day based on clinical prognosis [24, 26, 30, 33, 35]. About 80% of the included studies with available information on daptomycin dose started with a dose of 6 mg/kg/day, and only three studies used higher doses of 8–10 mg/kg/day, 9.2 mg/kg/day, or 10 mg/kg/day [5, 32, 35]. The exposure to the comparator vancomycin was based on receiving vancomycin for at least 48 hours after MRSA blood culture and remaining on vancomycin for at least 72 hours. Ten studies reported mean vancomycin trough levels within 12–20 mg/L (Table 1). However, Usery et al. reported vancomycin trough levels below the recommended value of 15 mg/L in 35% of the vancomycin arm [31]. Nine studies included patients who received additional anti-MRSA agents other than vancomycin. This ranged from 4.5% to 91% of patients in the vancomycin arm and 1.2% to 37% of patients in the daptomycin arm [26–28, 30, 32, 33, 36–38]. The proportion of patients with other added anti-MRSA antibiotics were comparable between vancomycin and daptomycin arms, except for the Fowler et al. study where 91% of the vancomycin-treated patients had combination therapy, whereas none of the daptomycin patients received combination therapy [33]. A similar study was excluded because it was from the same population [40].

The primary outcome, all-cause mortality, was measured after patients were followed-up for differing lengths of time. Thirteen studies reported 30-day mortality, four reported 60-day mortality, eight reported in-hospital mortality, two reported 14-day mortality, and one reported 42-day mortality. Other reported outcomes included clinical failure and persistent bacteremia/microbiological failure as defined by the authors (Table 1).

### Quality assessment

Thirteen out of 18 observational studies were of moderate to high quality based on the NOS quality scale, with scores of 6 to 8 points [25–31, 36–39, 41, 42]. The remaining observational studies scored less than 6 points on NOS scale (S1 Table in S2 File) [5, 32, 34, 35, 43, 44]. One of the two RCTs had a high risk-of-bias in the overall bias assessment (S1 Table in S2 File) because it was terminated before the intended study period due to low enrollment [24]. The other RCT (Randomized Control Trials) was of low risk of bias for most domains and high-risk-of-bias in the overall assessment because it had a high-risk-of-bias in the domain called intended intervention [33].

### Quantitative synthesis

#### Pooled all-cause mortality

**Fig 2:**
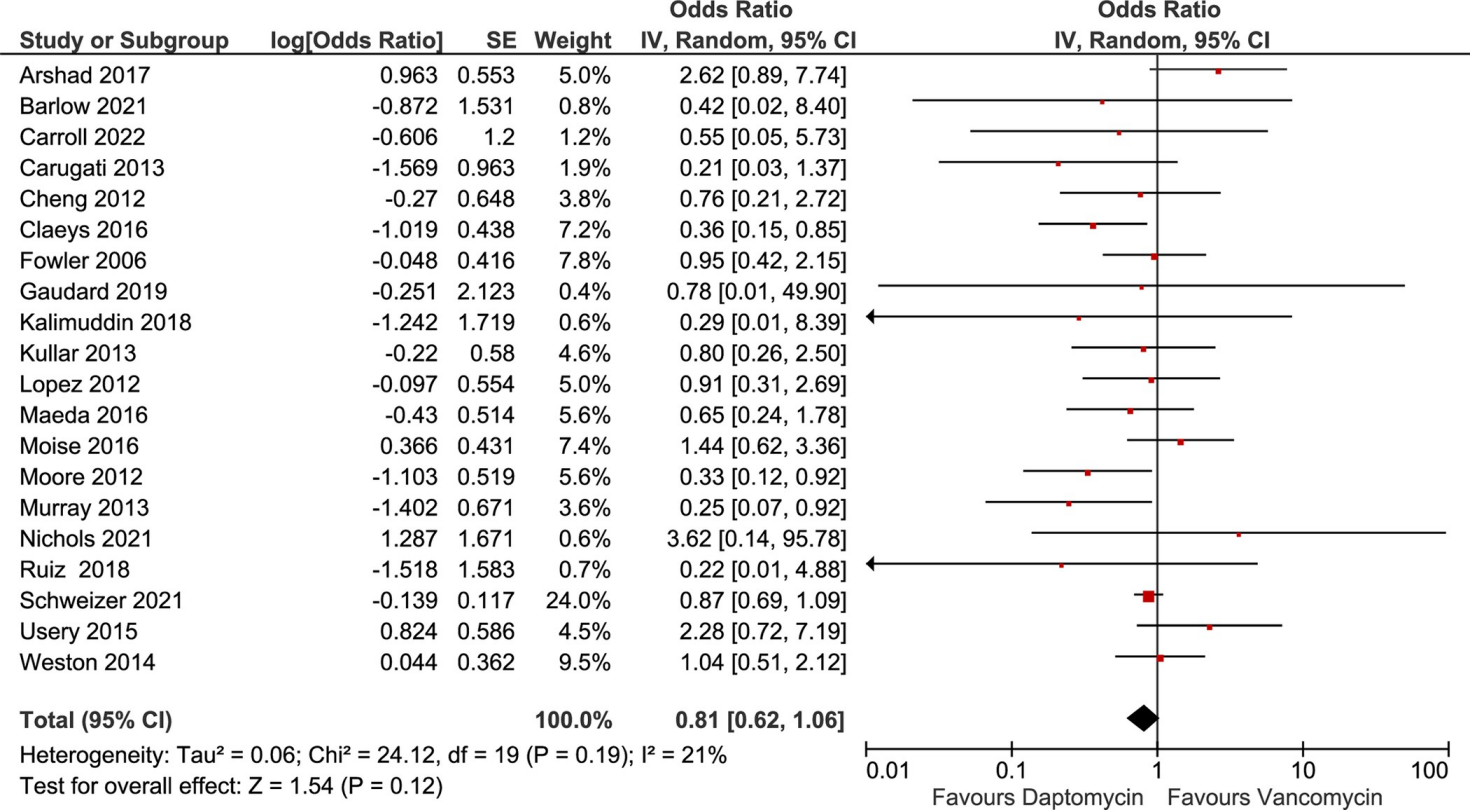
Forest plots of all included studies comparing the effects of daptomycin versus vancomycin on prevention of any reported all-cause mortality primary outcome.

The analysis of 20 included studies demonstrated that the overall pooled odds of all-cause mortality were 19% lower among those who received daptomycin compared to those who received vancomycin, although this difference was not statistically significant (OR = 0.81; 95% CI: 0.62, 1.06) Fig 2 and Table 2). The I^2^ value from pooling all studies was 21% (p = 0.12), suggesting low heterogeneity [45–47] (Table 2). Removing very small studies (*i*.*e*., involving less than 10 patients per arm) from the meta-analysis did not change the pooled odds of all-cause mortality (OR = 0.86; 95% CI, 0.70, 1.08) (S2 Table in S2 File). Sub-analyses were conducted based on variables that are relevant to clinical practice, including vancomycin MICs, switching time from one anti-MRSA antibiotic to another, follow-up duration before death, endocarditis, and using additional antibiotics [16, 21].

**Table 2:** Subgroup analysis and heterogeneity results for mortality. Summarized results of subgroup analysis of all-cause Mortality based daptomycin switch time, endocarditis, use of additional anti-MRSA agent, and vancomycin MIC.

Strata	Studies (n = 20)	Pooled OR (95% CI)	Heterogeneity	
			I^2^, %	P-value
Mortality by switch time to DAP				
Initial administered agent	4	0.48 (0.21, 1.11)	34	0.21
Switch within 3 days	5	0.45 (0.29, 0.69)	0	0.72
Initial/switch within 3 days	9	0.47 (0.33, 0.66)	0	0.56
Switch within 5 days	8	0.55 (0.36, 0.83)	24	0.24
Initial/switch within 5 days	12	0.53 (0.38, 0.76)	20	0.25
Switch after 5 days/any time	5	0.87 (0.70, 1.08)	0	0.50
Switch after 5 days/any time/no data	8	1.11 (0.76, 1.63)	31	0.17
VAN MIC cut off used to enroll patients				
VAN MIC ≥1 mg/L	11	0.60 (0.36, 0.99)	53	0.02
VAN MIC 1 to 2 mg/L	4	0.29 (0.17, 0.50)	0	0.99
Excluded VAN MIC ≥2 mg/L	16	0.70 (0.51, 0.97)	31	0.12
Studies used any VAN MIC or NR	9	0.90 (0.74, 1.11)	0	0.57
Mortality by follow-up duration				
In-hospital mortality	8	0.91 (0.56, 1.48)	6	0.38
14-day mortality	2	0.76 (0.36, 1.59)	0	0.66
30-day mortality	13	0.77 (0.52, 1.16)	48	0.03
42-day/60-day mortality	5	0.72 (0.40, 1.29)	33	0.20
Mortality by Endocarditis				
Included endocarditis patients	16	0.76 (0.54, 1.07)	48	0.02
Without endocarditis patients	4	0.58 (0.21, 1.58)	0	0.87
Use of additional anti-MRSA agent				
Some patients received added other anti-MRSA agent	9	0.65 (0.43, 0.96)	43	0.08
Excluded any patients added anti-MRSA	5	1.54 (0.61, 3.88)	0	0.64
No data on additional anti-MRSA agent/NR	6	0.81 (0.41, 1.61)	56	0.04

Sub-analysis of 9 studies that either used daptomycin as a first-line agent or switched to daptomycin within 3 days after positive blood culture demonstrated statistically significant 53% lower odds of mortality among the daptomycin group compared to the vancomycin group (I^2^ = 0%). Similarly, switching to daptomycin within 5 days was associated with significantly decreased mortality (Table 2 and S1 Fig 1S in S1 File). However, switching any time during treatment was not statistically significantly associated with decreased all-cause mortality (OR = 0.87; 95% CI, 0.70, 1.08) (Table 2 and S1 Fig 1S in S1 File). The stratified analysis of mortality based on the duration of follow-up did not change the results (Table 2 and S1 Fig 2S in S1 File).

**Fig 3:**
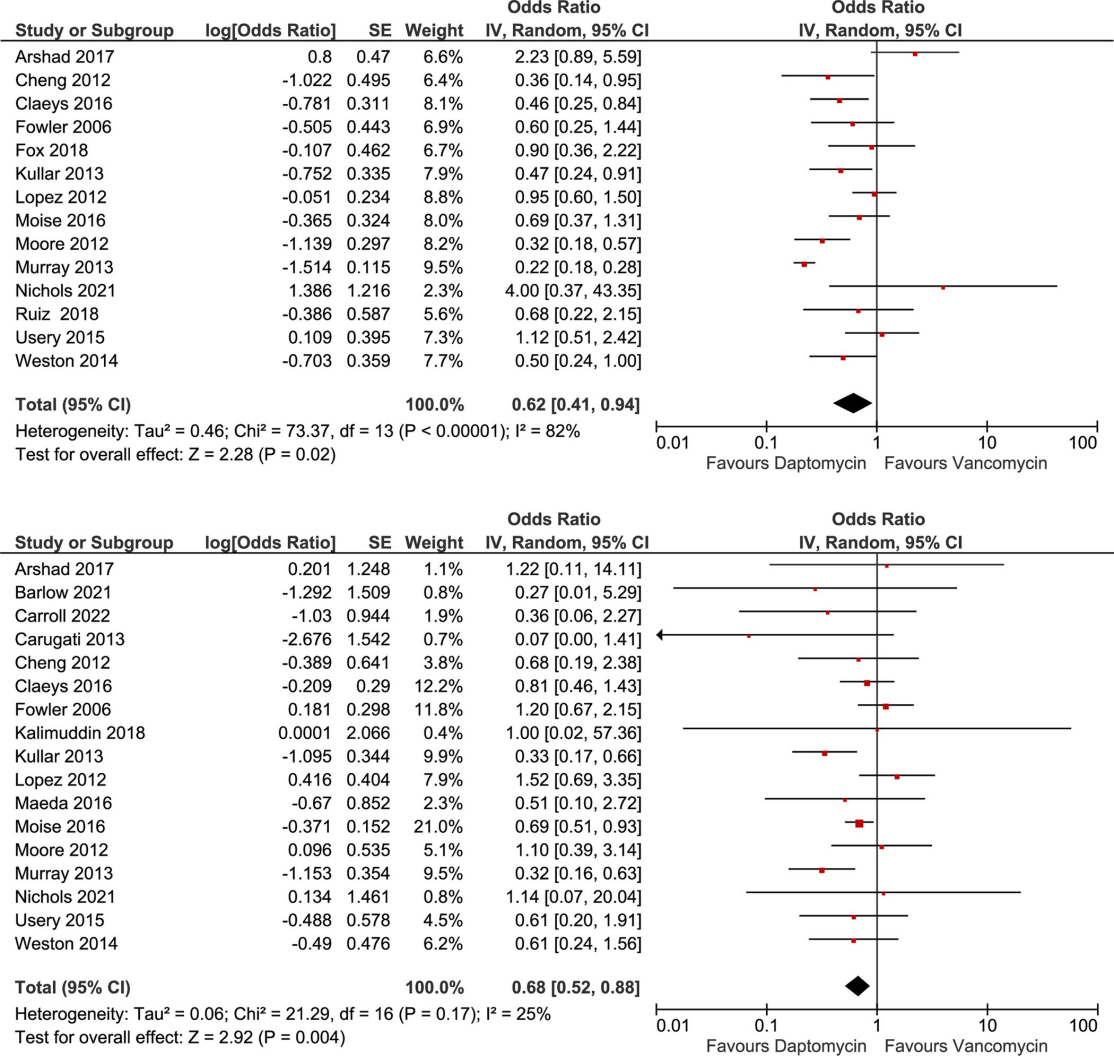
Forest plots of all included studies comparing the effects of daptomycin versus vancomycin on prevention of clinical failure (A) and persistent bacteremia (B) secondary outcomes.

Further subgroup analysis was performed based on vancomycin MIC levels of the infecting MRSA strains. Pooling studies that restricted enrollment to only patients with MRSA strains with vancomycin MIC ≥1 showed a significant 40% lower odds of mortality among daptomycin users compared to vancomycin users (OR = 0.60; 95% CI: 0.36, 0.99). Similarly, pooling studies that restricted enrollment to patients with MRSA strains with vancomycin MIC of 1 to 2 mg/L was associated with 71% lower odds of mortality among daptomycin users compared to vancomycin users (OR 0.29; 95% CI: 0.17, 0.50). Next, a sub-analysis of studies that excluded patients with MRSA strains with vancomycin MICs >2 resulted in significant 30% lower odds of mortality in daptomycin treated patients compared to non-daptomycin treatment (OR = 0.70; 95% CI: 0.51, 0.97) (Table 2). However, the lower odds of mortality associated with the daptomycin group disappeared among studies that included patients with any VAN MIC (Table 2 and S1 Fig 3S in S1 File).

A sub-analysis of the 9 studies that used additional anti-MRSA antibiotics together with vancomycin or daptomycin revealed lower odds of mortality among the daptomycin treated patients compared to vancomycin (OR = 0.65; 95% CI, 0.43, 0.96), with I^2^ = 43% (Table 2 and S1 Fig 4S in S1 File). The remaining sub-analyses yielded non-significant results (S1 Figs 5S and 6S in S1 File).

#### Clinical failure

When pooling the 14 studies that evaluated clinical failure, daptomycin use was significantly associated with 38% lower odds of clinical failure (OR = 0.62; 95% CI, 0.41, 0.94) (Fig 3A). The I^2^ value was 82% (p <0.01), suggesting high heterogeneity. Stratified analysis based on switch time indicated significant associations. Switching to daptomycin within 3 days and switching within 5 days were significantly linked with 64% and 61% decreased odds of clinical failure, compared to staying on vancomycin (Table 3). However, switching treatment to daptomycin at any time after 5 days of treatment did not yield a significant reduction in clinical failure compared to staying on vancomycin (OR = 1.03; 95% CI, 0.66, 1.61). In contrast, the subgroup analysis based on the switch time failed to explain the heterogeneity observed (Table 3 and S2 Fig 1S in S1 File). In the sub-analysis of studies evaluating patients infected with MRSA strains exhibiting an MIC of ≥1 mg/L, daptomycin-treated patients had significantly reduced odds of clinical failure in comparison to those who received non-daptomycin treatments, akin to the observed effect on mortality rates.

**Table 3:** Pooled results of stratified subgroup analysis for secondary outcomes: Clinical failure and persistent bacteremia. Summarized results of subgroup analysis of Clinical Failure and Persistent Bacteremia outcomes based on daptomycin switch time, endocarditis, use of additional anti-MRSA agent, and vancomycin MIC.

	Clinical Failure Outcome				Persistent Bacteremia Outcome			
Strata	No. of studies (N = 14)	Pooled OR (95% CI)	Heterogeneity		No. of studies (N = 17)	Pooled OR (95% CI)	Heterogeneity	
			I^2^, %	P-value			I^2^, %	P-value
Switch time to DAP								
Initial administered agent	2	NA	NA	NA	3	1.09 (0.63, 1.88)	0	0.64
Switch within 3 days	3	0.36 (0.19, 0.66)	83	0.003	3	0.45 (0.24, 0.85)	65	0.06
Initial/switch within 3-days	5	0.42 (0.25, 0.72)	76	0.002	6	0.58 (0.34, 1.01)	61	0.03
Switch within 5 days	6	0.39 (0.25, 0.60)	75	0.001	5	0.54 (0.37, 0.80)	51	0.09
Initial/switch within 5-days	8	0.42 (0.29, 0.63)	71	0.001	8	0.62 (0.44, 0.88)	46	0.07
Switch any time/not stated	6	1.03 (0.66, 1.61)	40	0.14	8	0.83 (0.53, 1.30)	2	0.41
VAN MIC cut off								
VAN ≥ 1 mg /L	9	0.54 (0.33, 0.90)	86	<0.001	9	0.65 (0.46, 0.92)	43	0.08
VAN MIC 1 to 2 mg/L	3	0.35 (0.18, 0.68)	83	0.003	5	0.60 (0.39, 0.92)	25	0.25
Excluded VAN MIC high limit of 2mg /L (≥2)	10	0.61 (0.44, 0.85)	53	0.02	13	0.67 (0.46, 0.98)	42	0.06
Studies used any VAN MIC /No data	5	0.77 (0.48, 1.23)	19	0.29	7	0.79 (0.51, 1.24)	3	0.40
Endocarditis								
Included endocarditis patients	12	0.65 (0.41, 1.02)	85	<0.001	13	0.72 (0.50, 1.03)	49	0.02
Without endocarditis patients/NR	2	0.47 (0.22, 0.98)	0	0.41	NA	NA	NA	NA
Used Additional anti-MRSA								
Added other anti-MRSA	8	0.47 (0.30, 0.71)	76	0.0001	8	0.64 (0.44, 0.91)	51	0.05
Without other anti-MRSA/no data	6	0.92 (0.53, 1.60)	58	0.03	8	0.79 (0.50, 1.25)	0	0.49

Further sub-analysis of the 3 studies that enrolled patients with vancomycin MIC 1 to 2 mg/L found a significant association between daptomycin use and lower odds of clinical failure compared to vancomycin (OR = 0.35; 95% CI, 0.18, 0.68). In contrast, sub-analysis among studies the enrolled patients with any MIC resulted in a non-significant association between daptomycin use and clinical failure in MRSA BSI patients (OR = 0.77; 95% CI, 0.48, 1.23). However, the association became significant after removing all studies with MRSA strains that had vancomycin MIC ≥ 2 (Table 3 and S2 Fig 2S in S1 File). The remaining sub-analyses results are available in S2 Figs 3S and 4S in S1 File).

#### Persistent bacteremia

Pooling the 16 studies with information on persistent bacteremia showed that daptomycin use was significantly associated with 32% lower odds of persistent bacteremia compared to vancomycin use among MRSA BSI patients (OR = 0.68; 95% CI, 0.52, 0.88) with lower heterogeneity between the studies (I^2^ = 25%, p = 0.17) (Fig 3B). Similarly, findings from stratified analyses based on switch time demonstrated that switching to daptomycin within 3 or 5 days after initial blood culture or using daptomycin as the initial choice were significantly associated with lower odds of persistent bacteremia compared to remaining on vancomycin (Table 3 and S3 Fig 1S in S1 File). Switching to daptomycin any time after 5 days was not statistically associated with lower odds of persistent bacteremia (OR = 0.83; 95% CI, 0.53, 1.30, I^2^ = 2%) (Table 3). Similar findings were observed in the sub-analyses based on vancomycin MIC and the use of additional anti-MRSA agents as shown in Table 3 and S3 Fig 2S in S1 File. The remaining sub-analyses are available in the S3 Figs 3S–6S in S1 File.

#### Sensitivity analyses

A sensitivity analysis by the leave-one-out approach did not change the results of the overall pooled odds of all-cause mortality among MRSA BSI patients, except for removing the Arshad et al., study where the protective effect of daptomycin over vancomycin became statistically significant (OR = 0.78; 95% CI, 0.62, 0.99, I^2^ = 9%) (S2 Table in S2 File) [39]. This was similar for the clinical failure and persistent bacteremia outcome (S2 Table in S2 File).

#### Publication bias

There was no evidence of publication bias for the outcome mortality using Egger’s regression test (p = 0.970) and Rank correlation tests (p = 0.631). Similarly, the Egger’s and Rank correlation tests for publication bias were not statistically significant for clinical failure (p = 0.640 and p = 0.667) and persistent bacteremia (p = 0.546 and p = 0.205).

## Discussion

This meta-analysis compared the effectiveness of daptomycin versus vancomycin in preventing poor outcomes among patients with MRSA BSIs. MRSA BSIs remain difficult to treat and are associated with high mortality. The existing treatment options for MRSA BSI require more investigation to balance the therapeutic effectiveness with potential toxicities. Switching to daptomycin within the first 5 days of antibiotic therapy was associated with a significant reduction in the odds of mortality compared to remaining on vancomycin. Further, earlier switching may be better: switching within three days conferred lower odds of mortality than switching within five days. Yet, a late switch to daptomycin after five days or any time during treatment was not associated with reduced mortality. Comparable results were observed between early daptomycin switching time and reduced clinical failure and persistent bacteremia.

It was somewhat surprising that daptomycin use was not significantly associated with mortality among initial users compared to starting on vancomycin. However, there was a trend in which most included studies favored daptomycin versus vancomycin for the prevention of mortality. Indeed, the overall pooled results from this study closely aligned with a recently published meta-analysis by Maraolo et al. demonstrating comparable efficacy between daptomycin and vancomycin in preventing mortality associated with MRSA bloodstream infections [48]. This meta-analysis is unique in that it is the first to include the concept of switching time. The study results indicate that patients may benefit upon switching from vancomycin to daptomycin, regardless of the vancomycin MIC levels. The current IDSA (Infectious Disease Society of America) guidelines recommend switching from vancomycin to daptomycin when there is vancomycin treatment failure, especially if the vancomycin MIC is > 2 mg/L [8]. However, findings from this study suggest this switch should occur early during infection (i.e., within 3 to 5 days), which may be around the time the laboratory confirms the isolation of MRSA but potentially before vancomycin MIC is known. Further, daptomycin use was significantly associated with reduced odds of mortality, clinical failure, and persistent bacteremia for studies that included patients infected with MRSA strains with a vancomycin MIC ranging from 1 to 2 mg/L, which is considered susceptible. Therefore, physicians should prioritize switching to daptomycin within 3–5 days of treatment while still factoring in the vancomycin MIC in making the decision to switch. These results fully agree with a prior meta-analysis by Samura et al that focused exclusively on seven studies that included bacteremia patients with MRSA vancomycin MIC > 1 mg/L [49]. In all, this meta-analysis supports other research that found that clinical decisions to switch patients to daptomycin should not solely rely on the vancomycin MIC because other factors may play a role on patient outcomes [50–52].

Persistence of bacteremia and clinical failure are direct outcomes associated with antibiotic treatment and are on the causal pathway between antibiotic treatment and mortality. Persistent bacteremia is an important outcome to assess when comparing antibiotic effectiveness because it is associated with increased risk of metastatic spread of the infection and mortality. The IDSA recommends reevaluating treatment after persistent bacteremia for 7 days [8]. Clinical failure definitions include persistence, but also encompass lack of response to the antibiotic as measured by new or worsening signs and symptoms of infection. The goals of antibiotic treatment are to prevent persistent infection, clinical failure, and mortality. However, the significant findings of improved efficacy against clinical failure in the daptomycin-treated group were relatively counterbalanced by higher rates heterogeneity.

Use of daptomycin may overcome the limitations of vancomycin. These limitations include difficulty in dosing vancomycin, nephrotoxicity, and the prevalence of strains of MRSA that have reduced susceptibility to vancomycin (e.g., high vancomycin MIC) [7, 16, 53].

The beneficial effect of combination antibiotic therapy with daptomycin in this study supports previous studies that found that adding other anti-MRSA antibiotics to daptomycin results in clearance of persistent MRSA BSIs and no clinical benefits if the decision to include additional agent occurs late in the treatment course [54, 55]. This advantage could be associated with the synergistic effects of daptomycin with other antibiotics such as ceftaroline [41, 44, 56]. However, the roles of combination therapy from this study remain unclear and warrants further study.

This meta-analysis has limitations. First, the study’s findings may be influenced by the inability to obtain the detailed reasons behind switching from vancomycin to daptomycin. Only six studies reported switching because of vancomycin MIC values or treatment failure [27, 29, 30, 32, 38, 42]. Second, the majority of the selected studies included patients with MRSA strains with vancomycin MICs ≥1 mg /L. Thus, the external validity of this meta-analysis may be limited, because findings from this study may not be generalizable to populations with vancomycin MIC < 1. Also, the results may not be generalizable to patients with complex infections. Fewer than 30% of the patients included in this meta-analysis had endocarditis. Also, comprehensive data on treatment outcomes for individual patients with endocarditis could not be acquired from all patients in all included studies. Third, most included studies recommended daptomycin at an initial dose of 6 mg/kg/day and thus this meta-analysis cannot assess the impact of high doses of daptomycin (i.e., 8–10 mg/kg IV once daily). Lastly, the link between switching from vancomycin to daptomycin and mortality may be confounded by immortal treatment bias because patients who die early do not switch antibiotics. However, this would impact both early and later switching, yet the beneficial effect was seen solely among those who switched to daptomycin early during treatment.

In conclusion, this study’s findings show that an early switch from vancomycin to daptomycin within the first 5 days of treatment initiation was associated with lower odds of mortality, persistent bacteremia, and clinical failure. This clinical benefit was not seen when the switch occurred later. These results, coupled with adverse events associated with vancomycin use, such as nephrotoxicity, may further support early daptomycin switch over remaining on vancomycin for MRSA BSI; even for susceptible vancomycin strains (MIC range 1–2 mg/L). However, more RCTs and prospective studies are needed to investigate the causal association between switching to daptomycin and improved outcomes among MRSA BSIs patients.

## Supporting information

S1 File Supporting figures. (DOCX)

S2 File Supporting tables. (DOCX)

S3 File Search terms. (DOCX)

S4 File Meta-analysis of observational studies in epidemiology checklist. (DOC)
