# Supplementary material for: Comparative effectiveness of daptomycin versus vancomycin among patients with methicillin-resistant Staphylococcus aureus (MRSA) bloodstream infections: A systematic literature review and meta-analysis
Source: PLoS One. 2024 Feb 21;19(2):e0293423. doi: 10.1371/journal.pone.0293423 (PMC10881006; doi:10.1371/journal.pone.0293423)
Supplement: S1 File — (DOCX) [file pone.0293423.s001.docx]

**Daptomycin versus Vancomycin Among Patients with Methicillin-resistant *Staphylococcus aureus* (MRSA) Bloodstream Infections: A Systematic Literature Review and Meta-analysis**

1. **Primary outcome: Mortality**

**S1 Figure 1S. Subgroup analysis of Mortality outcome based on switch time to daptomycin.**

**S1 Figure 2S. Forest plot sub-grouped by duration of mortality follow up: in-hospital, 30-day, 42-day, and 60-day mortality.**


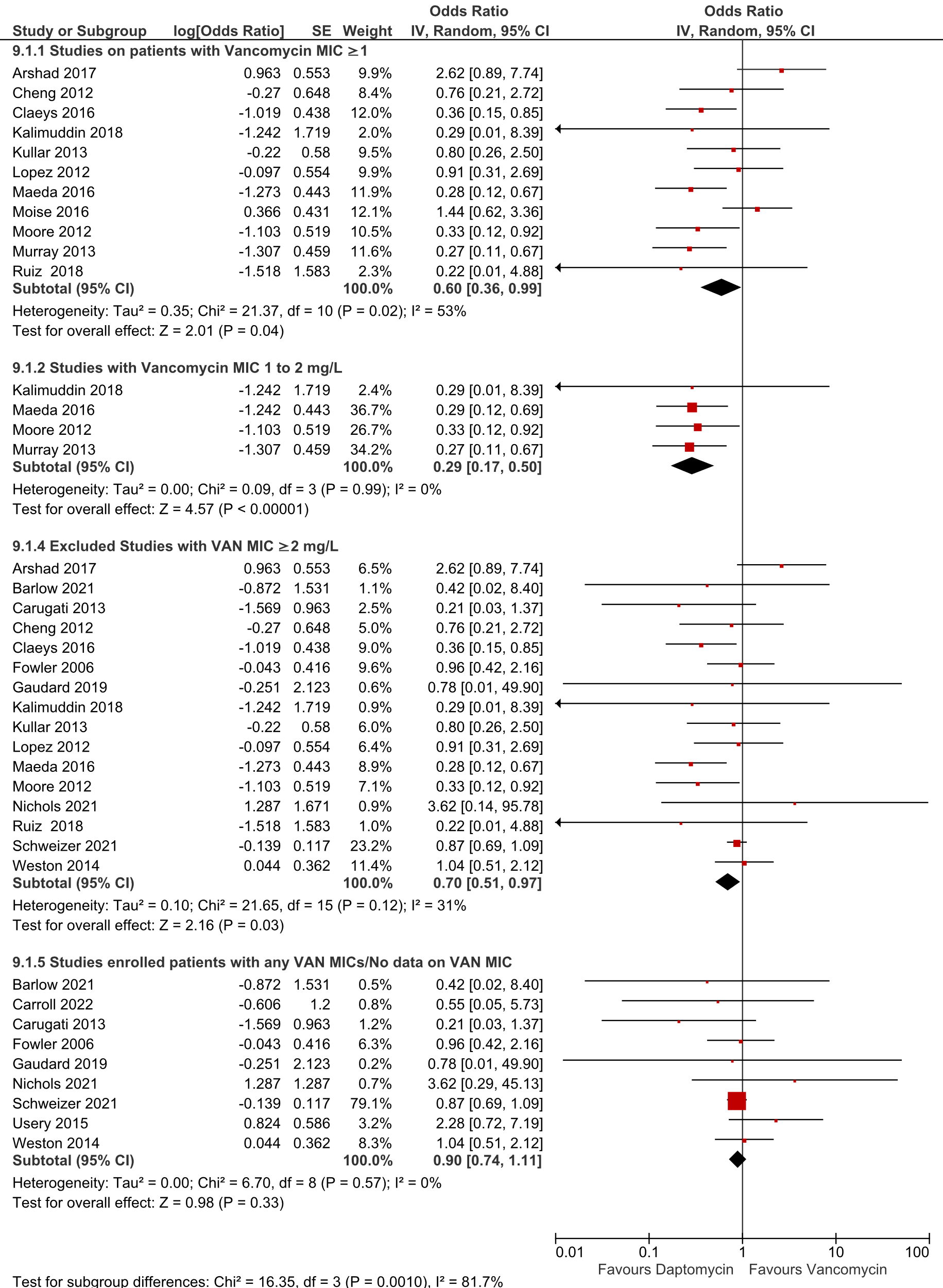


**S1 Figure 3S**. **Subgroup analysis of based on Vancomycin MIC on the association between daptomycin vs. vancomycin and mortality**

**S1 Figure 4S.** **Subgroup analysis of mortality based on treatment in combination with other anti-MRSA agents**

**S1 Figure 5S. Subgroup analysis of mortality based on inclusion of patients with endocarditis**


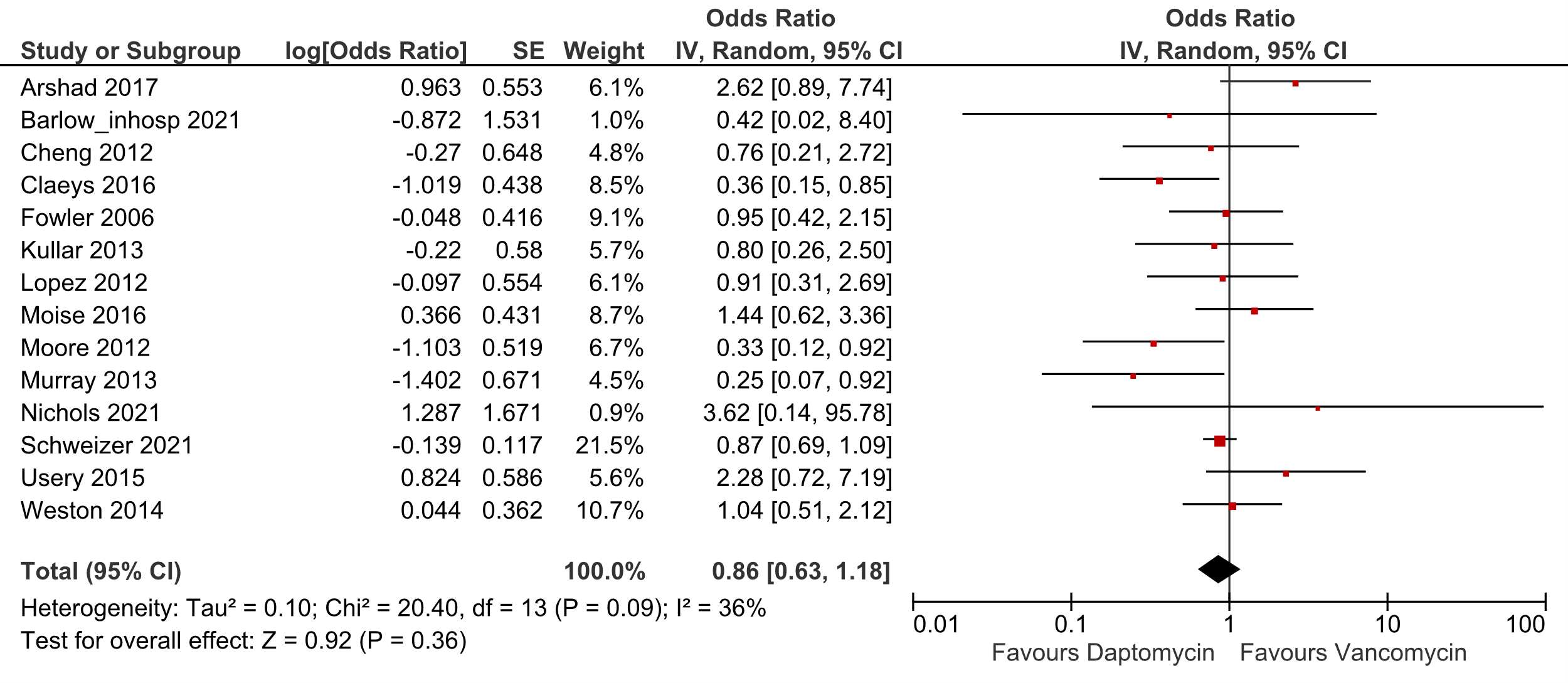


**S1 Figure 6S. Analysis of mortality after all small studies (n<10) were removed**

**2.Clinical Failure outcome**

**S2 Figure 1S. Clinical Failure meta-analysis based on switch time**


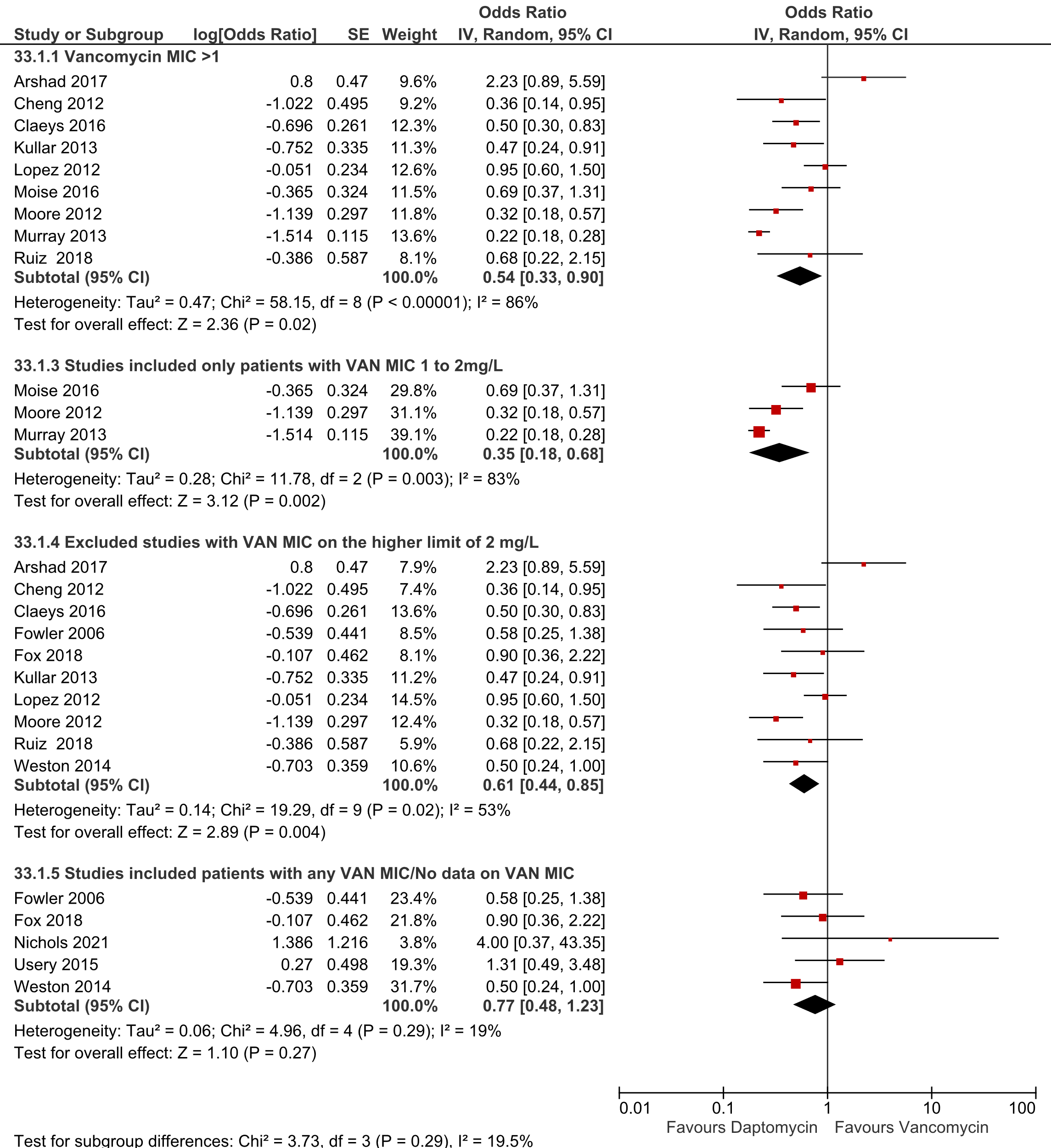


**S2 Figure 2S. Clinical Failure meta-analysis based on vancomycin MIC**

**S2 Figure 3S.** **Clinical Failure based on combination with other anti-MRSA antibiotics**


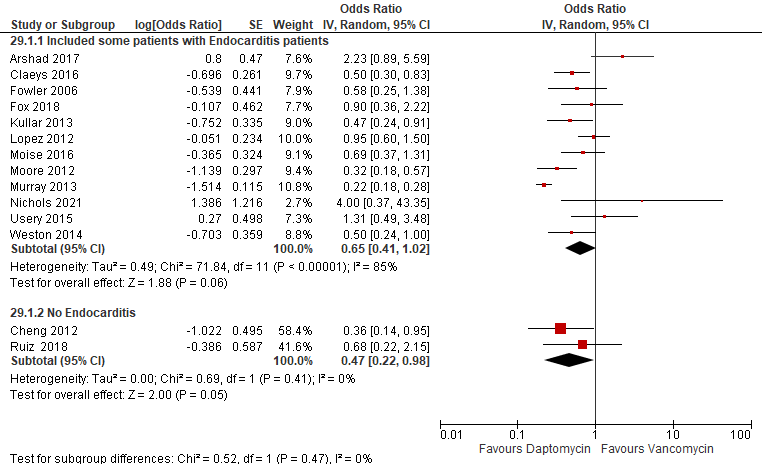


**S2 Figure 4S.** **Clinical Failure based on inclusion of endocarditis patients**

1. **Persistent Bacteremia outcome**

**S3 Figure 1S. Persistent Bacteremia based on switching time to daptomycin**


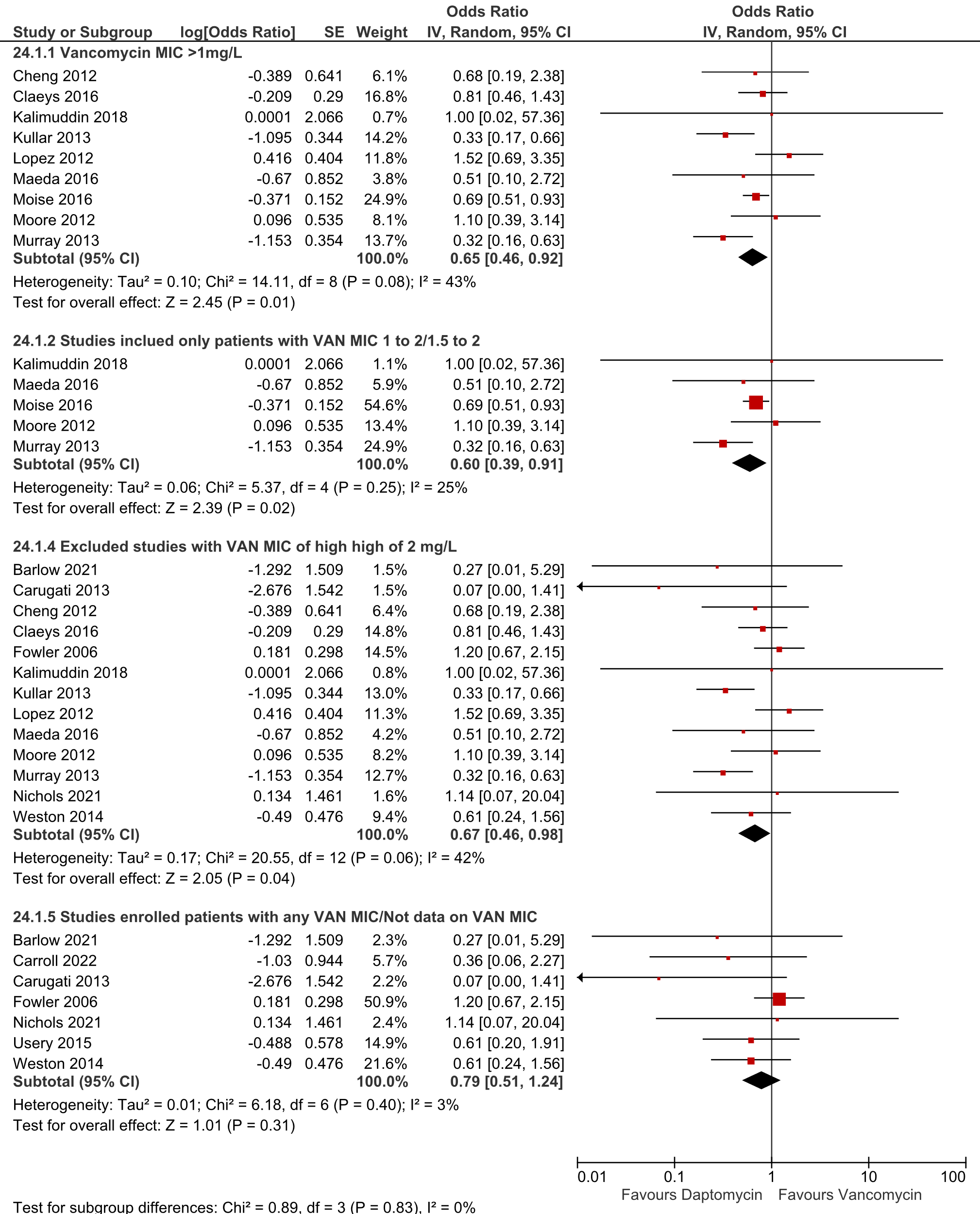


**S3 Figure 2S. Persistent Bacteremia based on Vancomycin MIC**

**S3 Figure 3S. Persistent Bacteremia among studies that included endocarditis patients**

**S3 Figure 4S. Persistent Bacteremia based on combination with other anti-MRSA antibiotic**


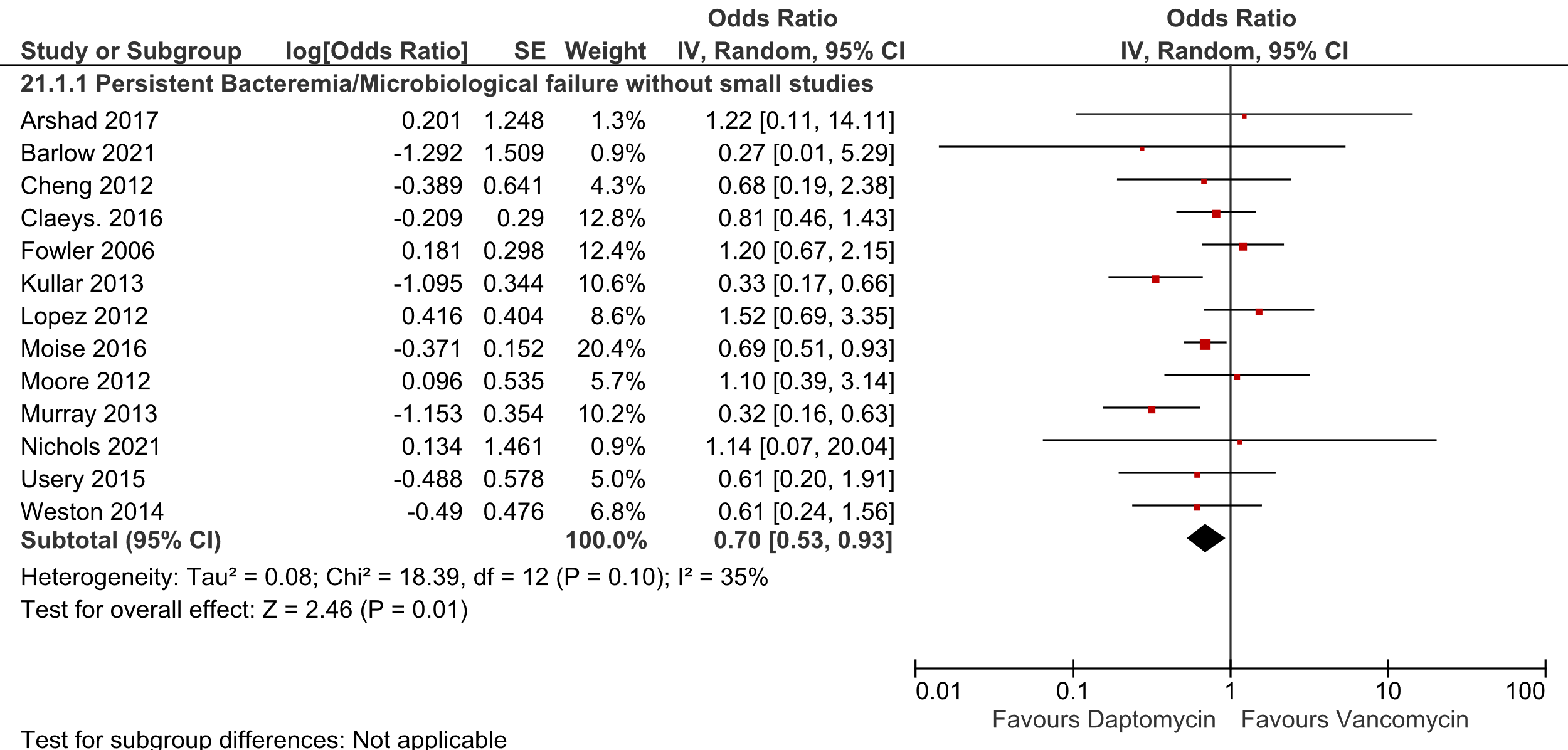


**S3 Figure 5S. Persistent Bacteremia removing studies with small sample sizes (n<10)**


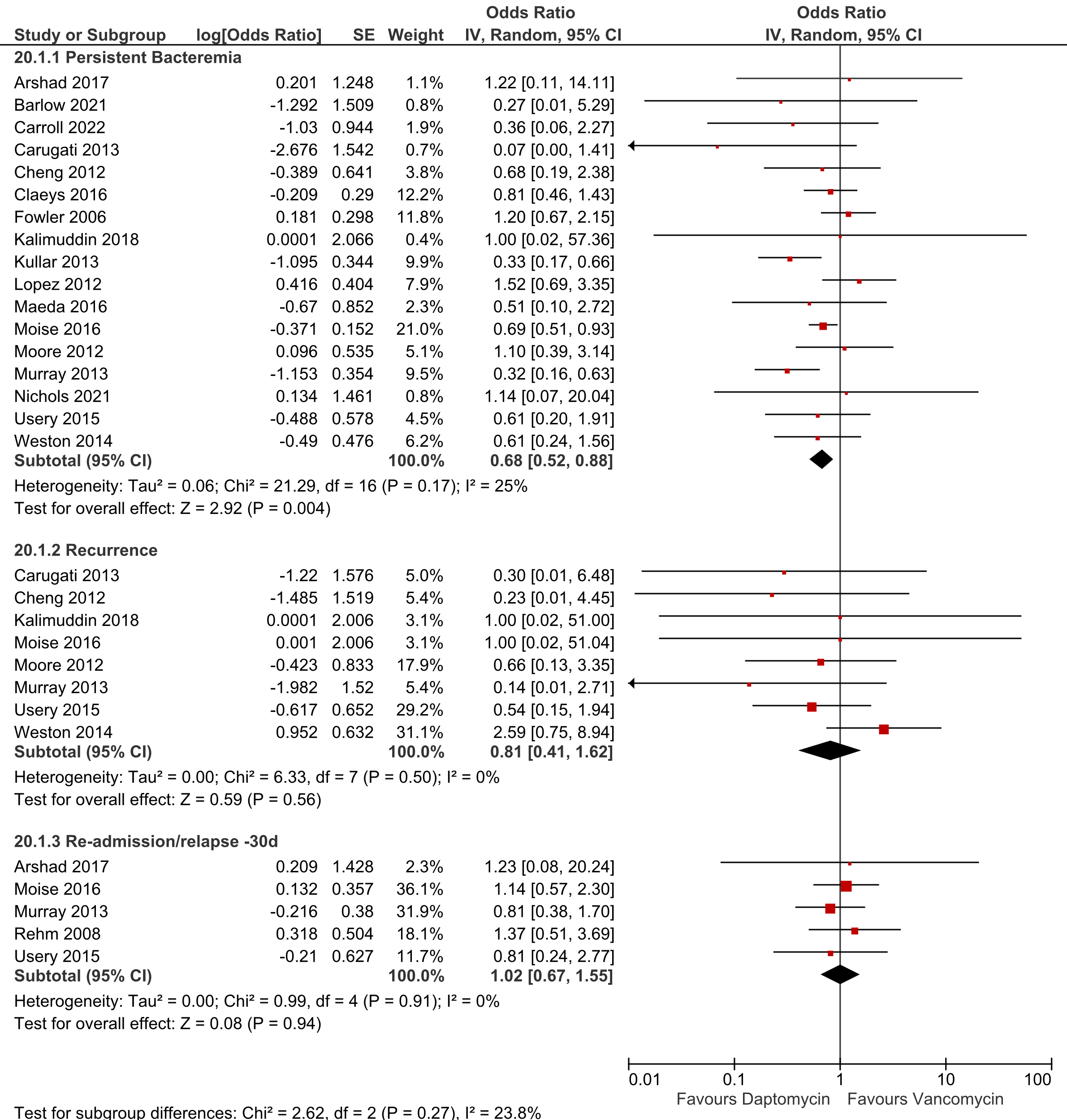


**S3 Figure 6S. Persistent Bacteremia based on types of microbial clearance**
