# Supplementary material for: Comparative effectiveness of daptomycin versus vancomycin among patients with methicillin-resistant Staphylococcus aureus (MRSA) bloodstream infections: A systematic literature review and meta-analysis
Source: PLoS One. 2024 Feb 21;19(2):e0293423. doi: 10.1371/journal.pone.0293423 (PMC10881006; doi:10.1371/journal.pone.0293423)
Supplement: S2 File — (DOCX) [file pone.0293423.s002.docx]

**Daptomycin Versus Vancomycin Among Patients with Methicillin-resistant Staphylococcus aureus (MRSA) Bloodstream Infections: A Systematic Literature Review and Meta-analysis**

**Supplementary information**

**Table S1.** Results of the critical quality appraisal of the included studies The Newcastle-Ottawa Scale (NOS) for assessing the quality of non-randomized studies in meta-analyses

| **Author, Year** | **Study design** | **Selection** | **Comparability** | **Outcome** | **Overall score** |  |
| --- | --- | --- | --- | --- | --- | --- |
| Moise et al., 2016 | Matched Cohort | +++ | **++** | +++ | 8 |  |
| Schweizer et al., 2021 | Retro Cohortm | +++ | **++** | +++ | 8 |  |
| Arshad et al., 2017 | Matched Cohort | +++ | **++** | +++ | 8 |  |
| Murray et al., 2013 | Matched Cohort | +++ | + | +++ | 7 |  |
| Claeys et al., 2016 | Matched Cohort | ++++ | **++** | +++ | 9 |  |
| Weston et al., 2014 | Matched Cohort | +++ | ++ | +++ | 8 |  |
| Moore et al., 2012 | Case-control | +++ | **++** | +++ | 8 |  |
| Barlow et al., 2021 | Retro Cohort | +++ | **-** | +++ | 6 |  |
| Gaudard et al., 2013 | Matched Cohort | +++ | - | ++ | 5 |  |
| Lopez et al., 2012 | multiple designs | ++ | **++** | ++ | 6 |  |
| Maeda et al., 2016 | Retro Cohort | +++ | + | ++ | 6 |  |
| Carugati et al., 2013 | Matched Cohort | +++ | - | ++ | 5 |  |
| Ruiz et al., 2018 | Retro Cohort | ++ | + | ++ | 5 |  |
| Cheng et al., 2012 | Matched Cohort | ++ | + | ++ | 5 |  |
| Usery et al., 2015 | Retro Cohort | +++ | **-** | +++ | 6 |  |
| Kullar et al., 2013 | Quasi Exp. | +++ | + | +++ | 7 |  |
| Nichols et al., 2021 | Retro Cohort | +++ | ++ | - | 7 |  |
| Carroll et al., 2022 | Retro Cohort | ++ | + | + | 4 | |
|  | Randomization | Intended intervention | Missing outcome data | Outcome Assessment | Reporting | Overall Risk |
| Fowler 2006 | RCT | HRB | LRB | LRB | LRB | HRB |
| Kalimuddin 2018 | RCT | HRB | LRB | HRB | HRB | HRB |

HRB; High Risk-of-bias, LRB; Low Risk-of-bias, Retro; retrospective, Exp; experiment, RCT; Randomized controlled trials.

| Table S2. Results of leave‐one‐out and leave-all-small studies-out meta‐analysis (random‐effects model). | | |
| --- | --- | --- |
| Outcome: Mortality | | |
| Study excluded (First author) | Pooled OR (95% CI) | Heterogeneity (I^2^), % |
| None of the included | **0.81 (0.62, 1.06)** | 21 |
| Schweizer et al., | 0.78 (0.56, 1.09) | 25 |
| Arshad et al., | 0.78 (0.62, 0.99) | 9 |
| Murray et al., | 0.85 (0.67, 1.09) | 13 |
| Claeys et al., | 0.87 (0.68, 1.11) | 11 |
| Cheng et al., | 0.81 (0.61, 1.07) | 25 |
| Moore et al., | 0.86 (0.67, 1.10) | 13 |
| Moise et al., | 0.77 (0.59, 1.02) | 20 |
| Weston et al., | 0.78 (0.59, 1.05) | 24 |
| Kalimuddin et al., | 0.81 (0.62, 1.01) | 24 |
| Fowler et al., | 0.79 (0.59, 1.06) | 25 |
| Gaudard et al., | 0.81 (0.61, 1.06) | 25 |
| Maeda et al., | 0.82 (0.62, 1.09) | 25 |
| Usery et al., | 0.78 (0.61, 1.01) | 15 |
| Ruiz et al., | 0.82 (0.62, 1.07) | 23 |
| Barlow et al., | 0.81 (0.62, 1.07) | 25 |
| Carugati et al., | 0.83 (0.64, 1.08) | 18 |
| Lopez et al., | 0.80 (0.60, 1.07) | 25 |
| Kullar et al., | 0.81 (0.61, 1.07) | 25 |
| Nichols et al., | 0.80 (0.61, 1.05) | 23 |
| Carroll et al., | 0.81 (0.62, 1.07) | 25 |
| All small studies with n<10 | **0.86 (0.63, 1.18)** | 36 |
| **Outcome: Clinical failure** | | |
| Study excluded | Pooled OR (95% CI) | Heterogeneity (I^2^), % |
| None of the included | **0.62 (0.41, 0.94)** | 82 |
| Arshad et al., | 0.56 (0.37, 0.83) | 80 |
| Murray et al., | 0.66 (0.49, 0.90) | 50 |
| Claeys et al., | 0.64 (0.40, 1.00) | 84 |
| Cheng et al., | 0.64 (0.41, 1.00) | 84 |
| Moore et al., | 0.66 (0.42, 1.04) | 84 |
| Moise et al., | 0.61 (0.39, 0.95) | 83 |
| Weston et al., | 0.63 (0.40, 0.99) | 84 |
| Fowler et al., | 0.62 (0.40, 0.96) | 83 |
| Usery et al., | 0.59 (0.38, 0.90) | 82 |
| Ruiz et al., | 0.61 (0.40, 0.95) | 83 |
| Fox et al. 2018 | 0.60 (0.39, 0.93) | 83 |
| Lopez et al., | 0.59 (0.39, 0.90) | 79 |
| Kullar et al., | 0.63 (0.40, 1.00) | 84 |
| Nichols et al., | 0.59 (0.39, 0.89) | 83 |
| All small studies with n<10 | **0.62 (0.41, 0.94)** | 83 |
| **Outcome: Persistent Bacteremia** | | |
| Study excluded | Pooled OR (95% CI) | Heterogeneity (I^2^), % |
| None of the included | **0.68 (0.52, 0.88)** | 25 |
| Arshad et al., | 0.67 (0.51, 0.88) | 29 |
| Murray et al., | 0.73 (0.59, 0.92) | 7 |
| Claeys et al., | 0.66 (0.49, 0.88) | 28 |
| Cheng et al., | 0.68 (0.51, 0.89) | 30 |
| Moore et al., | 0.66 (0.50, 0.87) | 27 |
| Moise et al., | 0.67 (0.48, 0.93) | 30 |
| Weston et al., | 0.68 (0.51, 0.90) | 29 |
| Kalimuddin et al., | 0.67 (0.51, 0.89) | 29 |
| Fowler et al., | 0.63 (0.49, 0.81) | 14 |
| Maeda et al., | 0.68 (0.52, 0.90) | 29 |
| Usery et al., | 0.68 (0.51, 0.90) | 29 |
| Barlow et al., | 0.68 (0.52, 0.89) | 28 |
| Carugati et al., | 0.69 (0.54, 0.89) | 21 |
| Lopez et al., | 0.64 (0.50, 0.81) | 13 |
| Kullar et al., | 0.73 (0.58, 0.93) | 9 |
| Nichols et al., | 0.67 (0.51, 0.88) | 29 |
| Carroll et al., | 0.68 (0.52, 0.90) | 28 |
| All small studies with n<10 | **0.70 (0.53, 0.93)** | 35 |
