# Supplementary material for: Comparative effectiveness of daptomycin versus vancomycin among patients with methicillin-resistant Staphylococcus aureus (MRSA) bloodstream infections: A systematic literature review and meta-analysis
Source: PLoS One. 2024 Feb 21;19(2):e0293423. doi: 10.1371/journal.pone.0293423 (PMC10881006; doi:10.1371/journal.pone.0293423)
Supplement: S3 File — (DOCX) [file pone.0293423.s003.docx]

**PubMed**

(

**"Methicillin-Resistant Staphylococcus aureus"[Mesh] OR**

**“**Methicillin-Resistant Staphylococcus aureus”[tw] OR

"Methicillin Resistant Staph aureus"[tw] OR

“MRSA”[tw] OR

“Staphylococcus aureus methicillin resistant”[tw] OR

“Staph aureus methicillin resistant”[tw]

)

**AND**

(

**"Bacteremia"[Mesh] OR**

“Bacteremia*”[tw] OR

“bacteraemia*”[tw] OR

“Bloodstream Infection*”[tw] OR

“Blood Stream Infection*”[tw] OR

“Staphylococcus aureus bacteremia*”[tw] OR

“staphylococcal bacteremia*”[tw] OR

“positive blood culture*”[tw] OR

“central line-associated bloodstream infection*”[tw] OR

“CLABSI”[tw]

)

**AND**

(

**"Daptomycin"[Mesh] OR**

"Daptomycin"[tw] OR

“Deptomycin”[tw] OR

“daptomycine”[tw] OR

“Cubicin”[tw] OR

“cidecin”[tw] OR

“dapcin”[tw] OR

“LY 146032”[tw] OR

“ly146032”[tw] OR

)

**AND**

(

**"Vancomycin"[Mesh] OR**

"Vancomycin"[tw] OR

“vanco*”[tw] OR

“Diatracin”[tw] OR

“adimicin”[tw] OR

“amplobac”[tw] OR

“balcorin”[tw] OR

“diatracin”[tw] OR

“edicin”[tw] OR

“firvanq”[tw] OR

“icoplax”[tw] OR

“ifavac”[tw] OR

“levovanox”[tw] OR

“lyphocin”[tw] OR

“maxivanil”[tw] OR

“norimko”[tw] OR

“selamat”[tw] OR

“vamysin”[tw] OR

“vanauras”[tw] OR

“vancam”[tw] OR

“vancamycin”[tw] OR

“varedet”[tw] OR

“voncon”[tw] OR

“vankomicin”[tw] OR

“vankomycin”[tw] OR

“vanmicina”[tw] OR

“vanococin”[tw] OR

“varedet”[tw] OR

“voncon”[tw] OR

“vondem”[tw]

)

(**"Methicillin-Resistant Staphylococcus aureus"[Mesh] OR “**Methicillin-Resistant Staphylococcus aureus”[tw] OR "Methicillin Resistant Staph aureus"[tw] OR “MRSA”[tw] OR “Staphylococcus aureus methicillin resistant”[tw] OR “Staph aureus methicillin resistant”[tw]) **AND** (**"Bacteremia"[Mesh] OR** “Bacteremia*”[tw] OR “bacteraemia*”[tw] OR “Bloodstream Infection*”[tw] OR “Blood Stream Infection*”[tw] OR “Staphylococcus aureus bacteremia*”[tw] OR “staphylococcal bacteremia*”[tw] OR “positive blood culture*”[tw] OR “central line-associated bloodstream infection*”[tw] OR “CLABSI”[tw]) **AND** (**"Daptomycin"[Mesh] OR** "Daptomycin"[tw] OR “Deptomycin”[tw] OR “daptomycine”[tw] OR “Cubicin”[tw] OR “cidecin”[tw] OR “dapcin”[tw] OR “LY 146032”[tw] OR “ly146032”[tw]) **AND** (**"Vancomycin"[Mesh] OR** "Vancomycin"[tw] OR “vanco*”[tw] OR “Diatracin”[tw] OR “adimicin”[tw] OR “amplobac”[tw] OR “balcorin”[tw] OR “diatracin”[tw] OR “edicin”[tw] OR “firvanq”[tw] OR “icoplax”[tw] OR “ifavac”[tw] OR “levovanox”[tw] OR “lyphocin”[tw] OR “maxivanil”[tw] OR “norimko”[tw] OR “selamat”[tw] OR “vamysin”[tw] OR “vanauras”[tw] OR “vancam”[tw] OR “vancamycin”[tw] OR “varedet”[tw] OR “voncon”[tw] OR “vankomicin”[tw] OR “vankomycin”[tw] OR “vanmicina”[tw] OR “vanococin”[tw] OR “varedet”[tw] OR “voncon”[tw] OR “vondem”[tw])

**2021-07-11 – PubMed – 274**

**2023-07-03 – PubMed - 309**

**Embase**

(

**'methicillin resistant Staphylococcus aureus'/exp OR**

**‘**Methicillin-Resistant Staphylococcus aureus’:ab,ti,kw OR

‘Methicillin Resistant Staph aureus’:ab,ti,kw OR

‘MRSA’:ab,ti,kw OR

‘Staphylococcus aureus methicillin resistant’:ab,ti,kw OR

‘Staph aureus methicillin resistant’:ab,ti,kw

)

**AND**

(

**'bacteremia'/exp OR**

‘Bacteremia*’:ab,ti,kw OR

‘bacteraemia*’:ab,ti,kw OR

‘Bloodstream Infection*’:ab,ti,kw OR

‘Blood Stream Infection*’:ab,ti,kw OR

‘Staphylococcus aureus bacteremia*’:ab,ti,kw OR

‘staphylococcal bacteremia*’:ab,ti,kw OR

‘positive blood culture*’:ab,ti,kw OR

‘central line-associated bloodstream infection*’:ab,ti,kw OR

‘CLABSI’:ab,ti,kw

)

**AND**

(

**'daptomycin'/exp OR**

‘Daptomycin’:ab,ti,kw,tn OR

‘Deptomycin’:ab,ti,kw,tn OR

‘daptomycine’:ab,ti,kw,tn OR

‘Cubicin’:ab,ti,kw,tn OR

‘cidecin’:ab,ti,kw,tn OR

‘dapcin’:ab,ti,kw,tn OR

‘LY 146032’:ab,ti,kw,tn OR

‘ly146032’:ab,ti,kw,tn

)

**AND**

(

**'vancomycin'/exp OR**

‘Vancomycin’:ab,ti,kw,tn OR

‘vanco*’:ab,ti,kw,tn OR

‘Diatracin’:ab,ti,kw,tn OR

‘adimicin’:ab,ti,kw,tn OR

‘amplobac’:ab,ti,kw,tn OR

‘balcorin’:ab,ti,kw,tn OR

‘diatracin’:ab,ti,kw,tn OR

‘edicin’:ab,ti,kw,tn OR

‘firvanq’:ab,ti,kw,tn OR

‘icoplax’:ab,ti,kw,tn OR

‘ifavac’:ab,ti,kw,tn OR

‘levovanox’:ab,ti,kw,tn OR

‘lyphocin’:ab,ti,kw,tn OR

‘maxivanil’:ab,ti,kw,tn OR

‘norimko’:ab,ti,kw,tn OR

‘selamat’:ab,ti,kw,tn OR

‘vamysin’:ab,ti,kw,tn OR

‘vanauras’:ab,ti,kw,tn OR

‘vancam’:ab,ti,kw,tn OR

‘vancamycin’:ab,ti,kw,tn OR

‘varedet’:ab,ti,kw,tn OR

‘voncon’:ab,ti,kw,tn OR

‘vankomicin’:ab,ti,kw,tn OR

‘vankomycin’:ab,ti,kw,tn OR

‘vanmicina’:ab,ti,kw,tn OR

‘vanococin’:ab,ti,kw,tn OR

‘varedet’:ab,ti,kw,tn OR

‘voncon’:ab,ti,kw,tn OR

‘vondem’:ab,ti,kw,tn

)

(**'methicillin resistant Staphylococcus aureus'/exp OR ‘**Methicillin-Resistant Staphylococcus aureus’:ab,ti,kw OR ‘Methicillin Resistant Staph aureus’:ab,ti,kw OR ‘MRSA’:ab,ti,kw OR ‘Staphylococcus aureus methicillin resistant’:ab,ti,kw OR ‘Staph aureus methicillin resistant’:ab,ti,kw) **AND** (**'bacteremia'/exp OR** ‘Bacteremia*’:ab,ti,kw OR ‘bacteraemia*’:ab,ti,kw OR ‘Bloodstream Infection*’:ab,ti,kw OR ‘Blood Stream Infection*’:ab,ti,kw OR ‘Staphylococcus aureus bacteremia*’:ab,ti,kw OR ‘staphylococcal bacteremia*’:ab,ti,kw OR ‘positive blood culture*’:ab,ti,kw OR ‘central line-associated bloodstream infection*’:ab,ti,kw OR ‘CLABSI’:ab,ti,kw) **AND** (**'daptomycin'/exp OR** ‘Daptomycin’:ab,ti,kw,tn OR ‘Deptomycin’:ab,ti,kw,tn OR ‘daptomycine’:ab,ti,kw,tn OR ‘Cubicin’:ab,ti,kw,tn OR ‘cidecin’:ab,ti,kw,tn OR ‘dapcin’:ab,ti,kw,tn OR ‘LY 146032’:ab,ti,kw,tn OR ‘ly146032’:ab,ti,kw,tn) **AND** (**'vancomycin'/exp OR** ‘Vancomycin’:ab,ti,kw,tn OR ‘vanco*’:ab,ti,kw,tn OR ‘Diatracin’:ab,ti,kw,tn OR ‘adimicin’:ab,ti,kw,tn OR ‘amplobac’:ab,ti,kw,tn OR ‘balcorin’:ab,ti,kw,tn OR ‘diatracin’:ab,ti,kw,tn OR ‘edicin’:ab,ti,kw,tn OR ‘firvanq’:ab,ti,kw,tn OR ‘icoplax’:ab,ti,kw,tn OR ‘ifavac’:ab,ti,kw,tn OR ‘levovanox’:ab,ti,kw,tn OR ‘lyphocin’:ab,ti,kw,tn OR ‘maxivanil’:ab,ti,kw,tn OR ‘norimko’:ab,ti,kw,tn OR ‘selamat’:ab,ti,kw,tn OR ‘vamysin’:ab,ti,kw,tn OR ‘vanauras’:ab,ti,kw,tn OR ‘vancam’:ab,ti,kw,tn OR ‘vancamycin’:ab,ti,kw,tn OR ‘varedet’:ab,ti,kw,tn OR ‘voncon’:ab,ti,kw,tn OR ‘vankomicin’:ab,ti,kw,tn OR ‘vankomycin’:ab,ti,kw,tn OR ‘vanmicina’:ab,ti,kw,tn OR ‘vanococin’:ab,ti,kw,tn OR ‘varedet’:ab,ti,kw,tn OR ‘voncon’:ab,ti,kw,tn OR ‘vondem’:ab,ti,kw,tn)

**2021-07-11 – Embase – 1092**

**2023-07-03 – Embase – 1246**

**Web of Science**

("Methicillin-Resistant Staphylococcus aureus" OR “Methicillin-Resistant Staphylococcus aureus” OR "Methicillin Resistant Staph aureus" OR “MRSA” OR “Staphylococcus aureus methicillin resistant” OR “Staph aureus methicillin resistant”)

AND

("Bacteremia" OR “Bacteremia*” OR “bacteraemia*” OR “Bloodstream Infection*” OR “Blood Stream Infection*” OR “Staphylococcus aureus bacteremia*” OR “staphylococcal bacteremia*” OR “positive blood culture*” OR “central line-associated bloodstream infection*” OR “CLABSI”)

AND

("Daptomycin" OR "Daptomycin" OR “Deptomycin” OR “daptomycine” OR “Cubicin” OR “cidecin” OR “dapcin” OR “LY 146032” OR “ly146032”)

AND

("Vancomycin" OR "Vancomycin" OR “vanco*” OR “Diatracin” OR “adimicin” OR “amplobac” OR “balcorin” OR “diatracin” OR “edicin” OR “firvanq” OR “icoplax” OR “ifavac” OR “levovanox” OR “lyphocin” OR “maxivanil” OR “norimko” OR “selamat” OR “vamysin” OR “vanauras” OR “vancam” OR “vancamycin” OR “varedet” OR “voncon” OR “vankomicin” OR “vankomycin” OR “vanmicina” OR “vanococin” OR “varedet” OR “voncon” OR “vondem”)

**2021-07-11 – Web of Science Core Collection – 512**

**2023-07-03 – Web of Science Core Collection - 589**

**Cochrane**

("Methicillin-Resistant Staphylococcus aureus" OR “Methicillin-Resistant Staphylococcus aureus” OR "Methicillin Resistant Staph aureus" OR “MRSA” OR “Staphylococcus aureus methicillin resistant” OR “Staph aureus methicillin resistant”)

AND

("Bacteremia" OR “Bacteremia*” OR “bacteraemia*” OR “Bloodstream Infection*” OR “Blood Stream Infection*” OR “Staphylococcus aureus bacteremia*” OR “staphylococcal bacteremia*” OR “positive blood culture*” OR “central line-associated bloodstream infection*” OR “CLABSI”)

AND

("Daptomycin" OR "Daptomycin" OR “Deptomycin” OR “daptomycine” OR “Cubicin” OR “cidecin” OR “dapcin” OR “LY 146032” OR “ly146032”)

AND

("Vancomycin" OR "Vancomycin" OR “vanco*” OR “Diatracin” OR “adimicin” OR “amplobac” OR “balcorin” OR “diatracin” OR “edicin” OR “firvanq” OR “icoplax” OR “ifavac” OR “levovanox” OR “lyphocin” OR “maxivanil” OR “norimko” OR “selamat” OR “vamysin” OR “vanauras” OR “vancam” OR “vancamycin” OR “varedet” OR “voncon” OR “vankomicin” OR “vankomycin” OR “vanmicina” OR “vanococin” OR “varedet” OR “voncon” OR “vondem”)

**2021-07-11 - 1 CochraneReview, 37 Trials - 38**

**2023-07-03 - 1 CochraneReview, 37 Trials - 38**
